# Immunotherapy utilization patterns in patients with advanced cancer and autoimmune disease

**DOI:** 10.1371/journal.pone.0300789

**Published:** 2024-04-16

**Authors:** Huaqi Li, Scott Huntington, Cary Gross, Shi-Yi Wang

**Affiliations:** 1 Department of Chronic Disease Epidemiology, Yale School of Public Health, New Haven, Connecticut, United States of America; 2 Cancer Outcomes, Public Policy, and Effectiveness Research (COPPER) Center, Yale Cancer Center, New Haven, Connecticut, United States of America; 3 Section of Hematology, Department of Internal Medicine, Yale School of Medicine, New Haven, Connecticut, United States of America; 4 Section of General Internal Medicine, Department of Internal Medicine, Yale School of Medicine, New Haven, Connecticut, United States of America; University of Oxford, UNITED KINGDOM

## Abstract

**Purpose:**

Immunotherapy has been shown to improve cancer survival, but there are no consensus guidelines to inform use in patients with both cancer and autoimmune disease (AD). We sought to examine immunotherapy utilization patterns between cancer patients with and without AD.

**Patients and methods:**

This retrospective cohort study utilized data from a de-identified nationwide oncology database. Patients diagnosed with advanced melanoma, non-small cell lung cancer, and renal cell carcinoma were included. Outcomes of interest included first-line immunotherapy, overall immunotherapy, and number of immunotherapy cycles. We used logistic and Poisson regression models to examine associations between AD and immunotherapy utilization patterns.

**Results:**

A total of 25,076 patients were included (796 with AD). Patients with AD were more likely to be female, White, receive care at academic centers, and have ECOG ≥ 3. Controlling for demographic and clinical variables, AD was associated with lower odds of receiving first-line (odds ratio [OR] = 0.68, 95% confidence interval [CI] 0.56–0.82) and overall (OR = 0.80, 95% CI 0.67–0.94) immunotherapy. Among patients who received at least one cycle of immunotherapy, there was no difference in mean number of cycles received between patients with and without AD (11.3 and 10.5 cycles respectively). The incident rate of immunotherapy cycles received for patients with AD was 1.03 times that of patients without AD (95% CI 1.01–1.06).

**Discussion:**

Patients with AD were less likely to receive immunotherapy as first-line and overall therapy for treatment of their advanced cancer. However, among those who did receive at least one cycle of immunotherapy, patients with AD received a similar number of cycles compared to patients without AD. This not only indicates that AD is not an absolute contraindication for immunotherapy in clinical practice but may also demonstrate overall treatment tolerability and net benefit in patients with AD.

## Introduction

Immunotherapy allows the immune system to detect and target tumor cells and has been shown to improve survival for patients with cancer [1–5]. However, evidence regarding immunotherapy use in patients with autoimmune disease (AD) is limited [6]. Because the immune systems of these patients are already prone to erroneously targeting healthy self-tissue as foreign tissue, there are concerns about side effects of immunotherapy among patients with AD [7]. Indeed, there exists no current consensus to guide the use of immunotherapy when caring for patients with AD [8], who comprise 2–30% of patients with cancer [8, 9]. To date, it is unclear whether patients with AD are less likely to receive immunotherapy during routine clinical care.

The decision of systemic therapy and immunotherapy is particularly complicated for patients with advanced cancer. As these patients may experience disease progression after first-line therapy, regimens can involve multiple lines of treatment. We hypothesize that patients with AD are less likely to receive immunotherapy as the first-line therapy, given physician hesitancy with regards to concerns of side effects and lack of current clinical guidelines [10]. However, a series of case reports has suggested that patients with both cancer and AD may respond just as well as patients without AD [11–16]. Considering that the benefits of immunotherapy for survival may outweigh the harms, patients with AD may instead receive immunotherapy as subsequent therapy. Thus, there may be no significant difference in receipt of overall immunotherapy between patients with and without AD.

Accordingly, we sought to determine the immunotherapy utilization patterns for patients with advanced cancer with and without AD. Specifically, we examined three immunotherapy use patterns, including immunotherapy as the first-line therapy, any immunotherapy use, and cycles of immunotherapy between the two groups. We examined cycles of immunotherapy as a proxy for treatment tolerability as well as to better understand clinician prescribing patterns. If patients with and without AD receive similar number of cycles of immunotherapy, then despite the potentially increased risk of side effects and toxicity, immunotherapy may be tolerated in select patients with AD. If treatment outcomes in patients with and without AD are similar, demonstrating that patients with AD are less likely to receive immunotherapy during routine-care compared to their counterparts without AD could highlight a need to increase utilization of immunotherapy in this at-risk population.

## Materials and methods

### Study design and data sources

This was a retrospective cohort study that used patient-level data from Flatiron Health’s nationwide de-identified electronic health record (EHR)-derived oncology database. Flatiron Health’s EHR-derived oncology database provides longitudinal, de-identified health record data abstracted from structured and unstructured information sources [17]. At the time of data collection, the database included approximately 280 cancer clinics across the United States [18]. Due to the nature of secondary analysis of fully anonymized data, this study received designation as non-human subjects research from the Yale University Institutional Review Board and thus the need for informed participant consent was waived. At no time during the study did authors have access to information that could identify individual participants.

### Cohort selection

**Fig 1:**
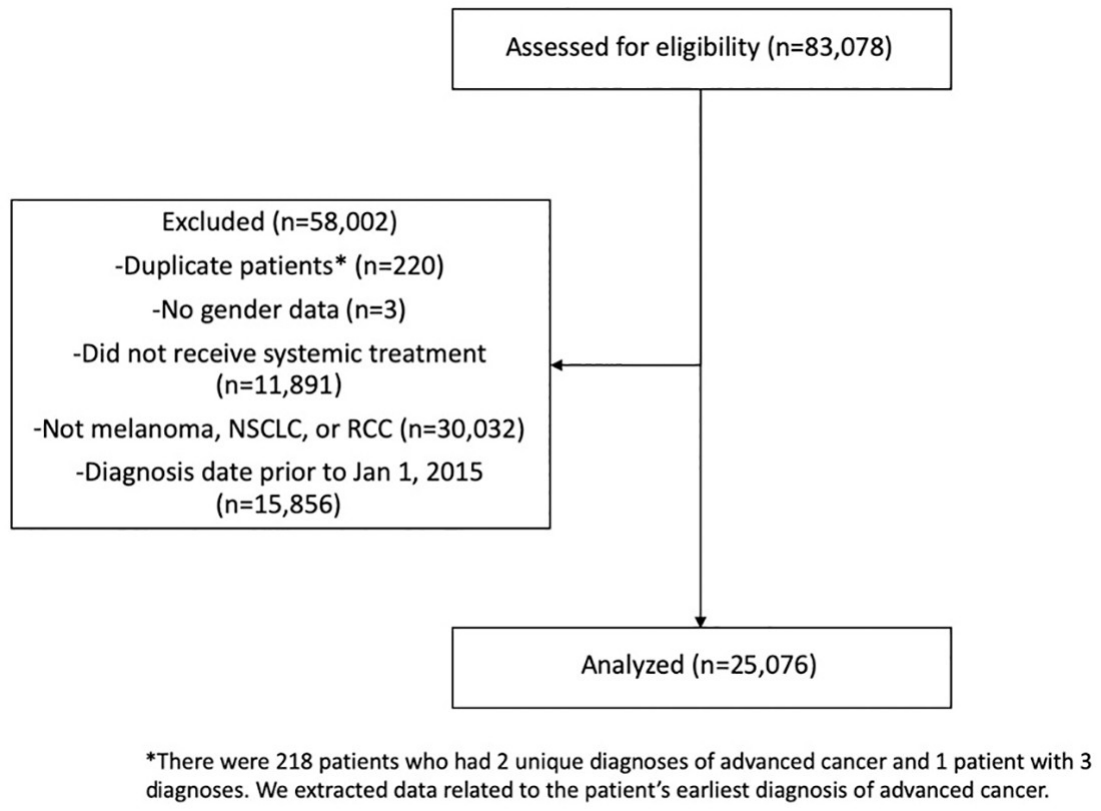
Patient flow diagram. NSCLC: Non-small cell lung cancer. RCC: Renal cell carcinoma.

Patients diagnosed with advanced cancers in which there was at least 1 FDA approved immunotherapy from January 1, 2015 to June 30, 2019 (date of data cut-off) were included in this analysis. A total of three cancer types were included: advanced melanoma, advanced non-small cell lung cancer (NSCLC), and metastatic renal cell carcinoma (RCC). Advanced disease was defined differently for each cancer site. For NSCLC, advanced disease was defined as stage IIIb or IV disease. For melanoma, advanced disease was defined as stage III or IV disease. For metastatic RCC, only stage IV disease was included. This data set excluded patients with a gap >90 days between diagnosis and first visit or medication order, with “visits” qualifying as any visit to a Flatiron Health oncology center. The 90-day cut-off is a standard data requirement in studies utilizing Flatiron data and serves to exclude incomplete data. This data set excluded deceased individuals i.e. Eastern Cooperative Oncology Group (ECOG) performance status values of 5. Only the primary cancer was included for patients with more than 1 diagnosis of advanced cancer. Patients were excluded if they did not receive any form of systemic therapy (i.e. immunotherapy and chemotherapy) or if they had missing data for gender (Fig 1).

### Outcome measures and covariates

Our outcomes of interest included immunotherapy as the first-line therapy (see S1a Fig), any immunotherapy use, and cycles of immunotherapy. Overall immunotherapy use indicates any instance of recorded immunotherapy use in the included patients with AD and advanced cancer during the timeframe of the study i.e. January 1, 2015 to June 30, 2019 (date of data cut-off). The key independent variable, AD, was created based on the International Classification of Diseases, Ninth and Tenth Revision (ICD-9, ICD-10, see S1 Table and S1b Fig). The database also includes clinical and demographic factors: baseline demographics included age, gender, race and, and smoking status. Clinical characteristics included year of diagnosis, cancer types, and the most recent ECOG performance status. We identified the insurance type, such as commercial health plan, Medicaid, Medicare, and others, as well as the practice type patients received care (community care vs. academic setting).

### Statistical analyses

All statistical analyses were performed using SAS University Edition (SAS Institute Inc, Cary, NC). Missing data were categorized as “unknown” in the analysis. Baseline demographics were analyzed using χ2 tests and t-tests for categorical and continuous variables, respectively. Patterns of first-line and overall immunotherapy utilization after diagnosis of advanced disease were assessed using logistic regression and the Wald χ2 test. Potential effect modification was assessed through interaction terms and stratified analyses. We also used Poisson regression to evaluate the relationship between number of cycles and AD status for the subset of patients receiving at least one cycle of immunotherapy in the overall cohort and for each of the three cancer types.

## Results

A total of 25,076 patients who were diagnosed with advanced cancer and treated with systemic therapy (i.e. immunotherapy and/or chemotherapy) between January 1, 2015, and June 30, 2019 were selected and included in this study. Of these patients, 796 (3.2%) had a diagnosis of AD. The most common ADs were rheumatoid arthritis (31.4%), pernicious anemia (10.6%), ulcerative enterocolitis (9.0%), psoriasis (5.7%), Crohn’s disease (5.3%), and multiple sclerosis (5.2%). Compared to those without AD, patients with AD were more likely to be female (50.3% vs 42.2%, p<0.001), be White (80.7% vs 71.9%, p<0.001), be on Medicare (21.9% vs 14.9%, p<0.001), have received care at an academic center (28.3% vs 8.2%, p<0.001), have advanced melanoma (17.5% vs 12.0%, p<0.001), and have an ECOG value of ≥3 (3.1% vs 2.8%, p = 0.001; Table 1).

**Table 1:** Baseline patient characteristics.

	Autoimmune Disease (n = 796)	No Autoimmune Disease (n = 24,280)	p-value
**Age (Mean)**	68	68	0.265^1^
**Advanced Cancer Diagnosis Year**			0.048^2^
**2015**	194 (24.4%)	5,560 (22.9%)	
**2016**	187 (23.5%)	5,807 (23.9%)	
**2017**	216 (27.1%)	5,861 (24.1%)	
**2018**	161 (20.2%)	5,419 (22.3%)	
**2019**	38 (4.8%)	1,633 (6.7%)	
**Gender**			<0.001^2^
**Female**	400 (50.3%)	10,242 (42.2%)	
**Male**	396 (49.8%)	14,038 (57.8%)	
**Race**			<0.001^2^
**White**	642 (80.7%)	17,444 (71.9%)	
**Black**	50 (6.3%)	1,869 (7.7%)	
**Asian**	7 (0.9%)	444 (1.8%)	
**Other**	41 (5.2%)	2,297 (9.5%)	
**Unknown**	56 (7.0%)	2,226 (9.2%)	
**Insurance Type**			<0.001^2^
**Commercial Health Plan**	332 (41.7%)	10,927 (45.0%)	
**Medicaid**	28 (3.5%)	977 (4.0%)	
**Medicare**	174 (21.9%)	3,622 (14.9%)	
**Other**	60 (7.5%)	2,834 (11.7%)	
**Unknown**	202 (25.4%)	5,920 (24.4%)	
**Practice Type**			<0.001^2^
**Academic**	225 (28.3%)	1,981 (8.2%)	
**Community**	571 (71.7%)	22,299 (91.8%)	
**Smoking status**			<0.001^2^
**History of smoking**	562 (70.6%)	18,624 (76.7%)	
**No history of smoking**	94 (11.8%)	2,681 (11.0%)	
**Unknown**	140 (17.6%)	2,975 (12.3%)	
**Cancer Type**			<0.001^2^
**Melanoma**	139 (17.5%)	2,913 (12.0%)	
**Non-Small Cell Lung Cancer**	609 (76.5%)	19,693 (81.1%)	
**Renal Cell Carcinoma**	48 (6.0%)	1,674 (6.9%)	
**ECOG Value**			0.001^2^
**0**	202 (25.4%)	7,107 (29.3%)	
**1**	262 (32.9%)	8,398 (34.6%)	
**2**	89 (11.2%)	2,890 (11.9%)	
**≥3**	25 (3.1%)	684 (2.8%)	
**Unknown**	218 (27.4%)	5,201 (21.4%)	

^1^T-test,

^2^Chi-squared test

### Immunotherapy utilization patterns

#### First-line immunotherapy

A total of 11,347 patients received first-line immunotherapy; 346 (43.5%) of the patients with AD and 11,001 (45.3%) of the patients without AD (p = 0.30). After controlling for age, year of advanced diagnosis, cancer type, gender, race, practice type, insurance status, ECOG value, and smoking status, AD was associated with lower odds of receiving first-line immunotherapy (odds ratio [OR] = 0.68, 95% confidence interval [CI] 0.56–0.82; Table 2). The interaction terms between practice type and AD (p = 0.34) as well as cancer type and AD (p = 0.34) were not statistically significant with regards to receipt of first-line immunotherapy.

**Table 2:** Logistic regression of covariates associated with immunotherapy use.

	First-line Immunotherapy		Overall Immunotherapy	
	OR	95% CI	OR	95% CI
**Age (Continuous)**	1.01	1.01–1.02	1.00	1.00–1.00
**Autoimmune Disease**				
**No**	1.00	Reference	1.00	Reference
**Yes**	0.68	0.56–0.82	0.80	0.67–0.94
**Advanced Cancer Diagnosis Year**				
**2015**	1.00	Reference	1.00	Reference
**2016**	1.72	1.54–1.92	1.30	1.20–1.41
**2017**	5.17	4.66–5.74	2.40	2.20–2.60
**2018**	9.95	8.95–11.06	4.28	3.90–4.70
**2019**	14.59	12.66–16.83	3.09	2.71–3.53
**Gender**				
**Male**	1.00	Reference	1.00	Reference
**Female**	1.07	1.00–1.14	1.04	0.98–1.11
**Race**				
**White**	1.00	Reference	1.00	Reference
**Black**	0.85	0.75–0.95	0.95	0.86–1.06
**Asian**	0.84	0.67–1.06	0.80	0.65–0.99
**Other**	1.00	0.89–1.11	1.09	0.99–1.21
**Unknown**	0.84	0.75–0.93	0.79	0.72–0.88
**Insurance Type**				
**Commercial Health Plan**	1.00	Reference	1.00	Reference
**Medicaid**	0.81	0.69–0.96	0.78	0.67–0.90
**Medicare**	1.02	0.93–1.12	0.99	0.90–1.08
**Other**	1.15	1.04–1.28	1.32	1.20–1.46
**Unknown**	1.11	1.03–1.21	0.98	0.91–1.06
**Practice Type**				
**Community**	1.00	Reference	1.00	Reference
**Academic**	1.49	1.31–1.68	1.39	1.24–1.56
**Smoking status**				
**No history of smoking**	1.00	Reference	1.00	Reference
**History of smoking**	0.85	0.77–0.95	0.94	0.86–1.04
**Unknown**	0.88	0.45–1.73	0.43	0.25–0.77
**Cancer Type**				
**Non-Small Cell Lung Cancer**	1.00	Reference	1.00	Reference
**Melanoma**	156.84	76.79–320.32	117.36	61.13–225.32
**Renal Cell Carcinoma**	75.60	58.99–96.90	22.26	16.62–29.81
**ECOG Value**				
**0**	1.00	Reference	1.00	Reference
**1**	1.02	0.95–1.11	0.93	0.87–1.01
**2**	1.06	0.95–1.18	0.82	0.74–0.90
**≥3**	1.30	1.08–1.58	0.68	0.57–0.81
**Unknown**	0.86	0.78–0.95	0.59	0.54–0.64

**Fig 2:**
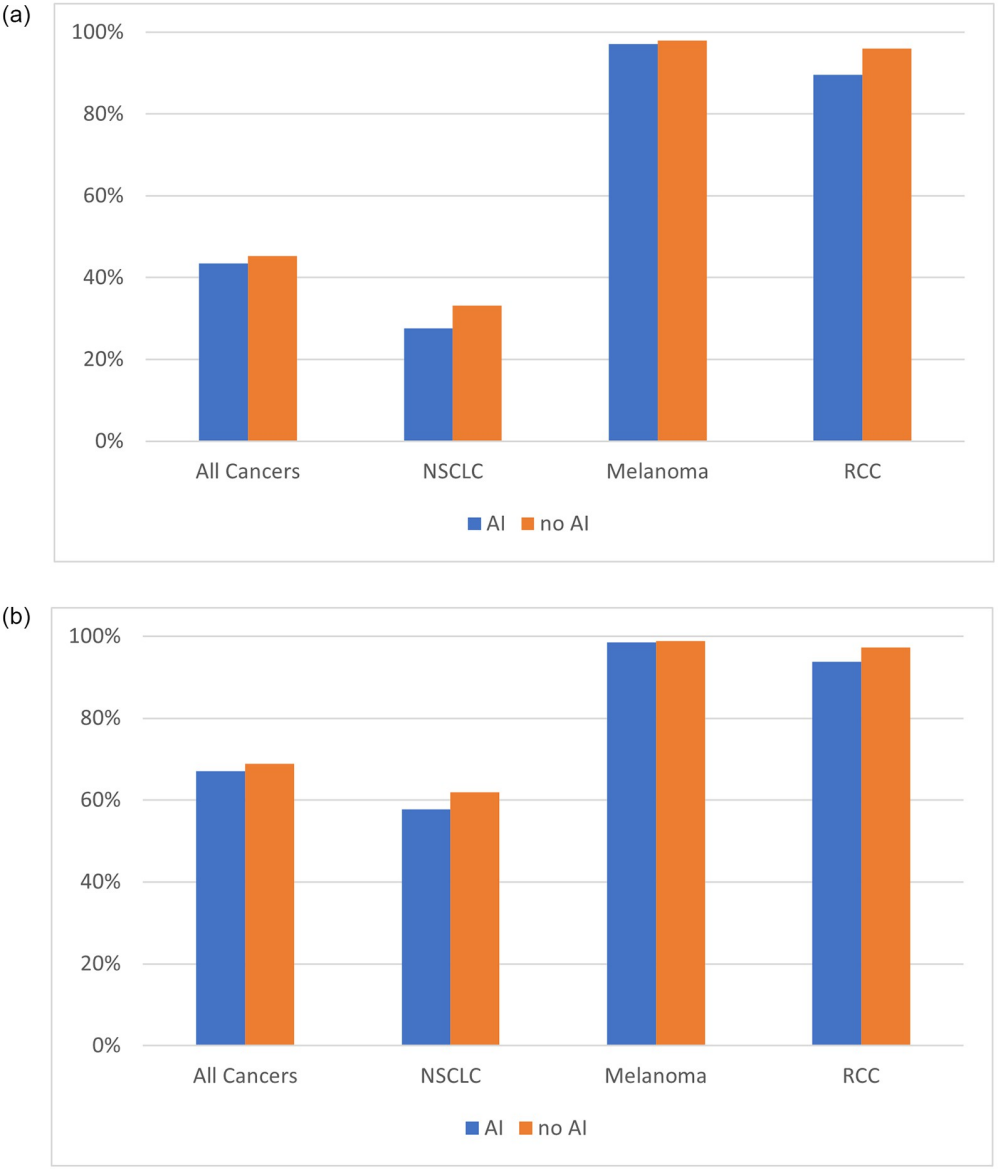
**a.** Percentage of patients receiving first-line immunotherapy by cancer type. NSCLC: Non-small cell lung cancer. RCC: Renal cell carcinoma. **b.** Percentage of patients receiving overall immunotherapy by cancer type. NSCLC: Non-small cell lung cancer. RCC: Renal cell carcinoma.

The stratified analysis for cancer type showed that first-line immunotherapy was received by the majority of patients (Fig 2a), both with and without AD, in advanced melanoma (97.1% and 98.0%, Fisher’s Exact p = 0.53) and metastatic RCC (89.6% and 96.0%, Fisher’s Exact p = 0.047). A lower percentage of patients, both with and without AD, received first-line immunotherapy in NSCLC (27.6% and 33.2%, p = 0.004). Fewer patients with AD received first-line immunotherapy compared to patients without AD in all three cancer types, with these differences being statistically significant in RCC (Fisher’s Exact p = 0.047) and NSCLC (p = 0.004). Controlling for demographic and clinical variables, patients with AD remained less likely to receive first-line immunotherapy in RCC (OR = 0.30, 95% CI 0.11–0.82) and NSCLC (OR = 0.69, 95% CI 0.56–0.84). There was no statistically significant difference in immunotherapy as the first line therapy between melanoma patients with and without AD.

#### Overall immunotherapy

A total of 17,242 patients received overall immunotherapy: 534 (67.1%) of the 796 patients with AD and 16,708 (68.8%) of the 24,280 patients without AD received overall immunotherapy, but this difference was not statistically significant (p = 0.30). Adjusted analyses revealed that AD was associated with lower odds of receiving overall immunotherapy (OR = 0.80, 95% CI 0.67–0.94; Table 2). The interaction effects between practice type and AD (p = 0.47) as well as cancer type and AD (p = 0.59) were not significant with regards to receipt of overall immunotherapy use.

The majority of patients with advanced melanoma and metastatic RCC, either with or without AD, received at least one cycle of immunotherapy; whereas approximately 60% of patients with NSCLC did (Fig 2b). Fewer patients with AD received overall immunotherapy compared to patients without AD in all three cancer types, with these differences being statistically significant in NSCLC (p = 0.038). Controlling for demographic and clinical variables, patients with AD remained less likely to receive overall immunotherapy in NSCLC (OR = 0.80, 95% CI 0.68–0.95).

#### Immunotherapy cycles

In the subset of patients who received at least one cycle of immunotherapy (n = 17,242), patients with AD received a mean of 11.3 cycles and a median of 7 cycles. Patients without AD received a mean of 10.5 cycles and a median of 6 cycles. Adjusting for age, year of advanced diagnosis, cancer type, gender, race, practice type, insurance status, ECOG value, and smoking status, the incident rate (IR) of immunotherapy cycles received for patients with AD was 1.03 times the IR for patients without AD (95% CI 1.01–1.06; Table 3).

**Table 3:** Poisson regression of covariates associated with number of immunotherapy cycles.

	Overall		NSCLC		Melanoma		RCC	
	IR	95% CI	IR	95% CI	IR	95% CI	IR	95% CI
**Autoimmune Disease**								
**No**	1.00	Reference	1.00	Reference	1.00	Reference	1.00	Reference
**Yes**	1.03	1.01–1.06	0.99	0.96–1.03	1.10	1.05–1.15	1.04	0.96–1.13

For patients with NSCLC, there was no statistically significant difference between the IR of immunotherapy cycles received for patients with and without AD (IR = 0.99, 95% CI 0.96–1.03). Similarly, immunotherapy cycles did not differ significantly among metastatic RCC patients with and without AD (IR = 1.04, 95% CI 0.96–1.13). However, for patients with melanoma, those with AD received a mean of 14.7 cycles (median = 10); whereas patients without AD received a mean of 13.0 cycles (median = 8). The IR of immunotherapy cycles received for patients with AD was 1.10 times the IR for patients without AD (95% CI 1.05–1.15).

## Discussion

While there is no consensus regarding immunotherapy use for patients with cancer and AD, the NCCN guidelines for NSCLC state that “single-agent immunotherapy or combination immunotherapy/chemotherapy regimens are not recommended if patients have contraindications to immunotherapy, which may include active or previously documented autoimmune disease” [19]. To our knowledge, there is no population study examining immunotherapy use among patients with cancer with and without AD. Our study provided the first empirical evidence that patients with AD in the USA were less likely to receive first-line and overall immunotherapy compared to patients without AD.

Our findings have several important clinical and policy implications. First, we observed differences in immunotherapy usage by cancer type: the vast majority of patients with metastatic RCC and melanoma received immunotherapy regardless of AD diagnosis whereas a lower percentage of patients with NSCLC received immunotherapy. In all three cancer types, fewer patients with AD received immunotherapy compared to those without AD. The high proportion of patients receiving both first-line and overall immunotherapy in melanoma and metastatic RCC is likely because these types of cancer have had a more established history of immunotherapy and such treatments are part of the current standard of care [20, 21]. On the other hand, immunotherapy, especially as first-line, is still a relatively new treatment modality in patients with NSCLC, so it is likely that fewer patients in these groups would be observed to receive immunotherapy.

Second, while patients with NSCLC did not receive immunotherapy as their first line therapy, approximately 30% of them received immunotherapy as the subsequent treatment. This difference may be due to the overall poorer prognosis of NSCLC compared to melanoma and RCC, with 5-year survival of NSCLC at 24%. Patients with NSCLC may have poorer initial clinical presentation compared to their melanoma and RCC counterparts, which compounded with the comorbidities of AD, may deter physicians from prescribing immunotherapy. However, after disease progression or recurrence, physicians may decide that the potential benefits of immunotherapy outweigh the potential risks of AD flare or immune-related adverse events (irAEs) such as thyroid dysfunction, dermatitis, and colitis in these patients. Research examining not only whether immunotherapy could be safely used for patients with AD but also whether delayed immunotherapy could achieve similar survival benefits is urgently needed.

Although patients with AD were less likely to receive immunotherapy compared to patients without AD, patients with AD received significantly more cycles of immunotherapy in the overall subset of patients who received at least one cycle of immunotherapy as well as specifically in patients with melanoma. Potentially, clinicians may be more likely to recommend immunotherapy to patients with AD who are healthier than their counterparts without AD or clinicians may have only recommended immunotherapy to patients with mild or well-controlled AD. Thus, these patients with AD may represent a subset of patients who are more able to withstand the side-effects associated with treatment. Alternatively, patients with AD may have better survival; thus, could receive more cycles of immunotherapy than those without AD. Literature regarding the association between AD and survival among patients with cancer is inconclusive. In female cancers such as breast, endometrial, and ovarian cancers, survival benefit was found in patients with AD [22]. However, in lung cancer, AD was not shown to influence overall survival [23]. Additionally, in cancers of the digestive tract, patients with AD had poorer prognosis and decreased survival [24]. Several recent studies have shown that in patients with AD receiving immunotherapy, survival outcomes were overall equivalent compared to those without AD and that disease flares and irAEs were largely mild [25–27]. Interestingly, an association between occurrence of irAEs and longer survival was found in patients with AD [25, 27]. This survival benefit may stem from the successful activation of T cells by immunotherapy which also manifests as immune-associated toxicity. However, numerically worse survival has also been documented in patients with disease flares on immunotherapy, although not statistically significant and potentially related to early cessation of therapy [26].

Our analyses have several limitations. First, this was a retrospective study and thus relied on pre-existing data. There may be misclassification of some patients with regards to AD status due to incomplete or incorrect ICD-9 and ICD-10 coding. However, we are confident in the overall quality and comprehensiveness of the data provided by Flatiron Health. In our analyses, we included all patients who had ever been diagnosed with an AD as we utilized pre-existing ICD coding. We were unable to specify the timeline in which these AD diagnoses were made (i.e. whether it was before or after the initiation of systemic therapy) as this data was physician recorded and could not be ascertained whether it referred to the date the AD was first diagnosed or the date that the diagnosis was entered into the EMR. This could introduce bias because AD is generally a contraindication for immunotherapy, so oncologists may be more likely to indicate a diagnosis of AD when they are considering immunotherapy. Future studies using site-specific data with access to date of diagnosis should evaluate the use of systemic therapy in pre-existing AD. In our analyses, we did not formally assess whether immunotherapy was administered as monotherapy or in combination with chemotherapy. We also did not formally assess the use of targeted therapies in our study population as we chose to focus on immunotherapy utilization given our research question regarding patients with AD. Inclusion of this information in future studies would further build upon the findings of this study and enhance our knowledge on systemic therapy utilization patterns in this patient population. Academic centers with multiple specialties may provide Flatiron Health with more complete EHR data compared to community centers, so differences in the percentage of patients with AD being treated at academic versus community centers may be a result of better reporting of ICD-9 and ICD-10 codes at academic centers and not actual patient differences. It is also unclear whether our data includes patients who were treated in other facilities for early stage disease and then subsequently received treatment for advanced disease at Flatiron Health-affiliated clinics. Additionally, only 3.1% of our study population had AD. Existing literature estimates 2–30% of cancer patients have concurrent AD, but prevalence may differ by cancer type. Also, the prevalence of AD among patients with cancer may differ by cancer types, and our study contained only patients with advanced cancer in which at least 1 immunotherapy regimen had been FDA approved. Future research validating claim codes for AD diagnosis is needed.

In conclusion, patients with coexisting cancer and AD were less likely to receive immunotherapy, overall and as first-line therapy. Taking into consideration the potential side effects of immunotherapy in this patient population, clinical practices should give thought to adopting immunotherapy regimens later on in the treatment pathway. The intersection between AD and cancer is understudied and more research in this area is needed to better understand the nuances observed in this study. We await the results of the recently initiated phase Ib nivolumab trial (ClinicalTrials.gov Identifier: NCT03816345) in this patient population to confirm our present findings.

## Supporting information

S1 Fig **a.** Types of immunotherapy first received by patients. **b.** Types of autoimmune disease.
(ZIP)

S1 Table ICD-9 and ICD-10 codes for autoimmune disease. (DOCX)

S1 File (DOCX)
