# Supplementary figures and images for: Immunotherapy utilization patterns in patients with advanced cancer and autoimmune disease
Source: PLoS One. 2024 Apr 16;19(4):e0300789. doi: 10.1371/journal.pone.0300789 (PMC11020359; doi:10.1371/journal.pone.0300789)

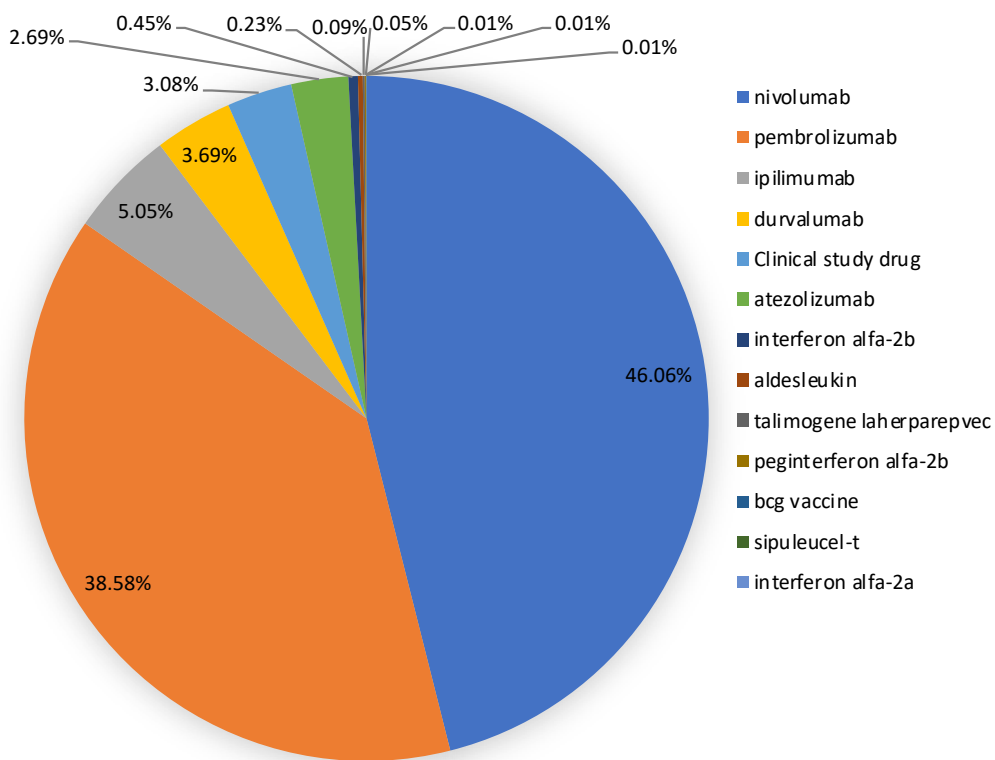

Supplement: S1 Fig — a. Types of immunotherapy first received by patients. b. Types of autoimmune disease. (ZIP) [file pone.0300789.s001.zip › S1a_Fig.pdf]

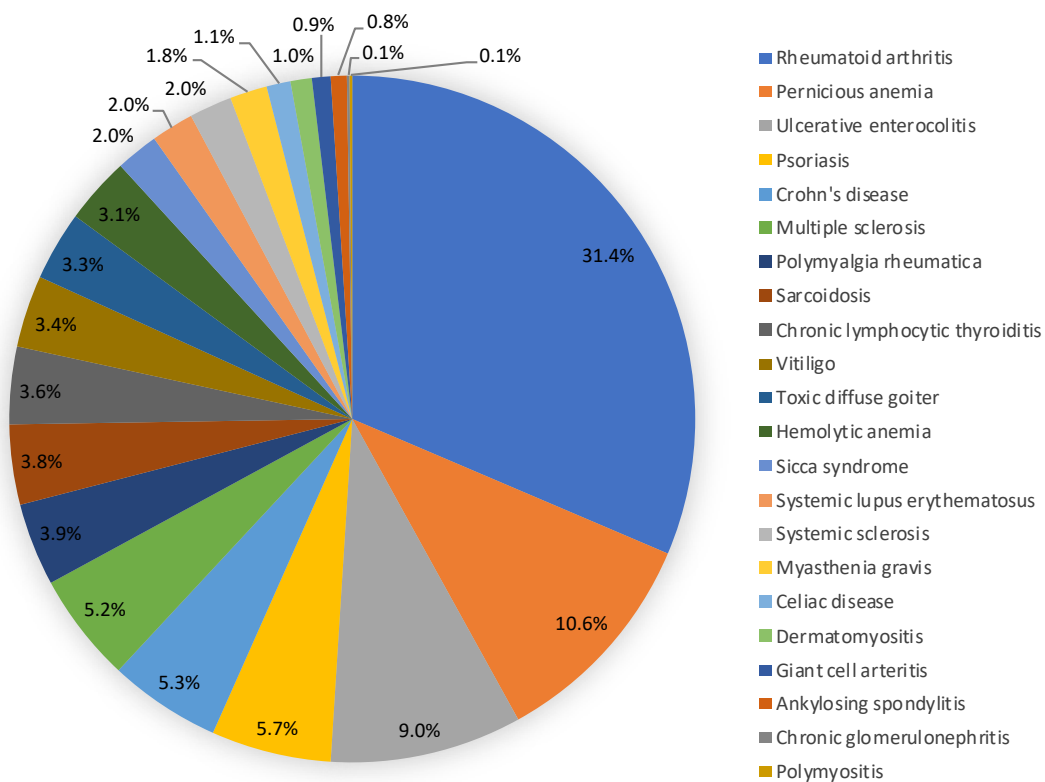

Supplement: S1 Fig — a. Types of immunotherapy first received by patients. b. Types of autoimmune disease. (ZIP) [file pone.0300789.s001.zip › S1b_Fig.pdf]
