# Supplementary material for: Immunotherapy utilization patterns in patients with advanced cancer and autoimmune disease
Source: PLoS One. 2024 Apr 16;19(4):e0300789. doi: 10.1371/journal.pone.0300789 (PMC11020359; doi:10.1371/journal.pone.0300789)
Supplement: S1 Table — (DOCX) [file pone.0300789.s002.docx]

Supplemental Table 1: ICD-9 and ICD-10 Codes for Autoimmune Disease

| **Disease** | **ICD-9** | **ICD-10** |
| --- | --- | --- |
| Acute glomerulonephritis | 580.4 | N01.3 |
| Ankylosing spondylitis | 720.0 | M45.9 |
| Behcet's syndrome | 136.1 | M35.2 |
| Celiac disease | 579.0 | K90.0 |
| Chagas disease | 086.0 086.2 | B57.0 B57.1 B57.2 |
| Chronic glomerulonephritis | 582.0 582.1 582.2 582.4 582.81 582.89 582.9 | N03.2 N03.3 N03.5 N03.8 N03.9 N08 |
| Chronic lymphocytic thyroiditis | 245.2 | E06.3 |
| Crohn's disease | 555.0 555.1 555.2 555.9 | K50.10 K50.00 K50.80 K50.90 |
| Dermatomyositis | 710.3 | M33.90 |
| Giant cell arteritis | 446.5 | M31.6 |
| Goodpasture's syndrome | 446.21 | M31.0 |
| Hemolytic anemia | 283.0 | D59.0 D59.1 |
| Multiple sclerosis | 340 | G35 |
| Myasthenia gravis | 358.00 358.01 | G70.01 G70.00 |
| Pernicious anemia | 281.0 | D51.0 |
| Polymyalgia rheumatica | 725.0 | M35.3 |
| Polymyositis | 710.4 | M33.20 |
| Psoriasis | 696.0 696.1 696.2 696.3 696.4 696.5 696.8 | L40.54 L40.59 L40.0 L40.1 L40.2 L40.3 L40.4 L40.8 L41.0 L41.1 L41.8 L42 L44.0 L30.5 L44.8 |
| Rheumatoid arthritis | 714.0 714.1 714.2 714.30 714.31 714.32 714.33 714.81 V82.1 | M05.10 M06.9 M05.00 M05.30 M05.60 M06.1 M08.00 M08.3 M08.40 Z13.828 |
| Sarcoidosis | 135 | D86.9 |
| Sicca syndrome | 710.2 | M35.01 M35.00 |
| Systemic lupus erythematosus | 710.0 | M32.10 |
| Systemic sclerosis | 710.1 | M34.0 M34.1 M34.9 |
| Toxic diffuse goiter | 242.00 242.01 | E05.00 E05.01 |
| Ulcerative enterocolitis | 556.0 556.1 556.2 556.3 556.4 556.5 556.6 556.8 556.9 | K51.80 K51.20 K51.30 K51.40 K51.50 K51.00 K51.80 K51.90 K59.31 |
| Vitiligo | 709.01 | L80 |
