# Supplementary material for: Immunotherapy utilization patterns in patients with advanced cancer and autoimmune disease
Source: PLoS One. 2024 Apr 16;19(4):e0300789. doi: 10.1371/journal.pone.0300789 (PMC11020359; doi:10.1371/journal.pone.0300789)
Supplement: S1 File — (DOCX) [file pone.0300789.s003.docx]

October 23, 2019

**NOT HUMAN SUBJECTS RESEARCH**

**Determination Date**: 10/23/2019

| **Investigator:** | Cary Gross |
| --- | --- |
| **Type of Review:** | Not Human Subject Research Determination |
| **Title of Study:** | Patterns of Care and Outcomes among Patients with Cancer in the Flatiron Health EHR-Derived Database |
| **IRB Protocol ID:** | 2000026765 |
| **Documents:** | • Flatiron protocol, Category: IRB Protocol; |

**Additional Information Regarding the Determination:**

The Yale IRB determined that the investigator is not engaged in research involving human subjects. As such, IRB review and approval are not required.

This determination applies only to the activities described in the submission and does not apply should any changes be made. If changes are being considered and there are questions about whether IRB review is needed, please contact the IRB Office.

**Important Reminder:**

- In conducting this activity, you should refer to and follow the Investigator Manual (HRP-103) as applicable, which can be found in the IRB Library within the IRB system.
